# Risk Factors for Postoperative Pneumonia: A Case-Control Study

**DOI:** 10.3389/fpubh.2022.913897

**Published:** 2022-07-08

**Authors:** Bingbing Xiang, Shulan Jiao, Yongyu Si, Yuting Yao, Feng Yuan, Rui Chen

**Affiliations:** ^1^Geriatric Diseases Institute of Chengdu/Cancer Prevention and Treatment Institute of Chengdu, Department of Anesthesiology, Chengdu Fifth People's Hospital (The Second Clinical Medical College, Affiliated Fifth People's Hospital of Chengdu University of Traditional Chinese Medicine), Chengdu, China; ^2^Department of Anesthesiology, The Second Affiliated Hospital of Kunming Medical University, Kunming, China; ^3^Department of Anesthesiology, The Second Affiliated Hospital of Chongqing Medical University, Chongqing, China

**Keywords:** postoperative pneumonia, perioperative, risk factors, pathogen distribution, outcomes

## Abstract

**Background:**

Postoperative pneumonia is a preventable complication associated with adverse outcomes, that greatly aggravates the medical expenses of patients. The goal of our study is to identify risk factors and outcomes of postoperative pneumonia.

**Methods:**

A matched 1:1 case-control study, including adult patients who underwent surgery between January 2020 and June 2020, was conducted in the Second Affiliated Hospital of Kunming Medical University in China. Cases included all patients developing postoperative pneumonia within 30 days after surgery, defined using consensus criteria. Controls were selected randomly from the matched eligible population.

**Results:**

Out of 17,190 surgical patients, 264 (1.54%) experienced postoperative pneumonia. Increased age, chronic obstructive pulmonary disease, emergency surgery, postoperative reduced albumin, prolonged ventilation, and longer duration of bed rest were identified as significant risk factors independently associated with postoperative pneumonia. Regarding prognostic implications, postoperative pneumonia was associated with longer length of hospital stay, higher ICU occupancy rate, higher unplanned re-operation rate, and higher in-hospital mortality rate. Postoperative pneumonia was most commonly caused by Gram-negative pathogens, and multidrug resistant bacteria accounted for approximately 16.99% of cases.

**Conclusions:**

Postoperative pneumonia is associated with severe clinical outcomes. We identified six independent risk factors that can aid in risk stratification and management of patients at risk of postoperative pneumonia, and the distribution of causative pathogens can also help in the implementation of effective interventions.

**Clinical Trial Registration:**

www.chictr.org.cn, identifier: chiCTR2100045986.

## Background

Every year, more than 300 million patients worldwide undergo surgery (1). Estimates of procedure-related mortality in surgical patients range from 1 to 4%, of which more than one-fifth are due to perioperative complications, with an incidence ranging from 3 to 16% (1, 2). Studies have shown (3) that almost half of perioperative complications can be effectively prevented, and the current incidence of permanent disability or death caused by these complications still accounts for 0.4% to 0.8%. Even with timely treatment, related complications will still reduce the long-term survival time of surgical patients.

Postoperative pneumonia (POP) is one most common complication of these and it is defined as hospital-acquired pneumonia or ventilator-associated pneumonia in post-surgical patients. Currently, postoperative pneumonia has the highest incidence of hospital-acquired pneumonia in the world, accounting for approximately 50% of all nosocomial pneumonias, with an incidence of 1.5 to 15.8% (4–7). Postoperative pneumonia can adversely affect the outcomes of surgical patients and may even threaten their lives. Mortality related to postoperative pneumonia among surgical patients has been reported to range from 20 to 50%, and the mortality rate varies by the type of surgery (8). Studies have shown that the fatality rate caused by postoperative pneumonia can be up to 9–50%, and even after risk adjustment, the patients' 5-year survival rate after surgery is reduced by 66% (9). Among the remaining survivors, there is also evidence that postoperative pneumonia adversely affects the patients' early postoperative recovery and late quality of life. In addition, postoperative pneumonia can significantly prolong the hospital stay of surgical patients and significantly increase their postoperative ICU occupancy rate, readmission rate, reoperation rate and mortality rate (8, 9), which greatly aggravate the burden of medical expenses of patients and leads to an average increase by approximately 2–10 times of additional medical expenses (5, 9).

Therefore, it is obviously worthwhile to identify the perioperative risk factors for postoperative pneumonia and investigate the distribution of causative bacteria. The result would suggest the measures for risk reduction through action on modifiable factors, or increase vigilance in the presence of non–modifiable conditions. The result of causative bacteria could also aid in selection of antibiotics for post-infection treatment especially considered against the worldwide escalation of infection caused by multidrug resistant microorganisms. Our primary aim was to identify perioperative risk factors and outcomes of postoperative pneumonia. Our secondary aim was to investigate the distribution of causative bacteria and surgical specialty.

## Methods

### Study Design and Participants

This study protocol was approved by the Institutional Ethics Committee of the Second Affiliated Hospital of Kunming Medical University (Kunming, China, approval number: PJ-2021-39). Informed consent was waived due to the retrospective design of the study. The study was registered in the Chinese Clinical Trial Registry (Clinical Trials identifier: ChiCTR2100045986). This study is a single-center retrospective 1:1 case-control study. From the hospital's complete electronic medical record “Donghua”, a total of 17,190 patients who underwent surgery from January 1, 2020 to June 31, 2020 were included. The case group included all adult patients who followed for hospital acquired pneumonia (HAP) occurrence for 30 days after surgery. Controls were matched by surgical specialty and randomly selected at 1:1 from the remaining surgical patients without pneumonia. Exclusion criteria were age under 18 years, procedures outside an operating room, patients already intubated, procedures related to postoperative complications of previous surgery, outpatient procedures (hospital stay <24 h), patient's medical records missing or inadequate.

### Diagnosis of Pneumonia

The US Centers for Disease Control definition of pneumonia was used (7). Two or more serial chest radiographs with at least one of the following (one radiograph is sufficient for patients with no underlying pulmonary or cardiac disease): (i) New or progressive and persistent infiltrates, (ii) consolidation, (iii) cavitation; and at least one of the following: (a) fever (>38°C) with no other recognized cause, (b) leucopenia (white cell count < 4 × 10^9^ liter^−1^) or leukocytosis (white cell count > 12 × 10^9^ liter^−1^), (c) for adults > 70 years old, altered mental status with no other recognized cause; and at least two of the following: (a) new onset of purulent sputum or a change in character of the sputum, or increased respiratory secretions, or increased suctioning requirements, (b) new onset or worsening cough, or dyspnea, or tachypnea, (c) rales or bronchial breath sounds, (d) worsening gas exchange (hypoxemia, increased oxygen requirement, increased ventilator demand).

### Data Collection

Perioperative data were collected retrospectively. Demographic factors, including age, sex, weight, height, body mass index (BMI) and factors assessing general condition [such as the Glasgow Coma Scale (GCS) and American Society of Anesthesiologists (ASA) classification], were recorded. Patients' past medical history, including smoking, drinking, hypertension, diabetes, malignancy, stroke, chronic obstructive pulmonary disease (COPD), coronary heart disease, liver disease and renal dysfunction was assessed. Laboratory measurements were reviewed as last values before operation or first values after operation, such as albumin, hemoglobin (Hb), blood urea nitrogen and creatinine levels. Factors associated with surgery, including surgical specialty, duration of surgery, type of surgery (scheduled or emergency), surgery period (day or night), were also evaluated. Intraoperative variables, including blood loss, red blood cells (RBC) transfusions, human albumin infusion, amount of liquid input and invasive procedure (such as radial artery cannulation, deep vein catheterization and gastric tube intubation) were recorded. Additionally, perioperative factors pertaining to the respiratory system, such as the duration of mechanical ventilation (duration until tracheal extubation) and duration of bed rest (duration until patients' first off-bed activity) were also evaluated. Causative bacteria and Multi-drug-resistance (MDR, defined as non-susceptibility to at least one agent in three or more antimicrobial categories) were recorded. To assess the prognostic implications of postoperative pneumonia, length of hospital stay, admission to the intensive care unit (ICU) and in-hospital mortality rates were reviewed.

### Statistical Analysis

In this study, the mean and standard deviation (x ± s) were used to represent the measurement data conforming to a normal distribution and a homogenous variance, and the independent sample *t*-test was used for comparisons between the case group and the control group. The median (interquartile range) was used to represent the measurement data with a non–normal distribution, and the two groups were compared by the rank-sum test. All enumeration data were represented by frequency and percentage, and the two groups were compared by X^2^ test. All statistically significant factors on univariate analysis were selected for inclusion in the multivariate regression analysis conducted by a binary logistic regression analysis model. Bivariate odds ratios (OR) and 95% confidence intervals (CI) were also estimated. *P* < 0.05 was considered statistically significant. All statistical analyses were performed using SPSS software (version 24, SPSS Inc., United States).

## Results

### Patient Characteristics

A total of 17,190 surgical patients were selected for the study, of which 264 patients were diagnosed with postoperative pneumonia. Overall, the incidence of postoperative pneumonia was 1.54% (264/17,190). Table 1 summarizes the operative frequency and the incidence of postoperative pneumonia in each surgical specialty. Among patients undergoing neurosurgery, the frequency (102/600) and incidence (17.00%) of postoperative pneumonia were much higher than the others, followed by the thoracic (8.67%), general and digestive (2.69%), cardiac and vascular (2.29%), and hepatobiliary (2.26%). The postoperative pneumonia rates of these five surgical specialty all exceeded 2%.

**Table 1:** Distribution of postoperative pneumonia in each surgical specialty.

**Surgical specialty**	**The operation frequency**	**Frequency of POP**	**Rate of POP**
Neurosurgery	600	102	17.00%
Hepatobiliary	2,305	52	2.26%
General and digestive	1,300	35	2.69%
Thoracic	300	26	8.67%
Urology	2,624	16	0.61%
Obstetric	1,450	8	0.55%
Traumatology	542	6	1.11%
Gynecology	593	4	0.67%
Cardiac and vascular	218	5	2.29%
Burns	357	4	1.12%
Ear–nose–throat (ENT)	456	2	0.44%
Neurology	367	2	0.54%
Orthopedic	851	1	0.12%
Other	2,227	0	0%

### Causative Pathogens

The distribution of pathogenic bacteria is shown in Table 2. A total of 153 different strains of pathogens were isolated from the sputum specimens of patients with postoperative pneumonia by coculture. Among which 98 isolates (64.05%) were Gram-negative bacteria, 42 isolates (27.45%) were Gram-positive bacteria, and 13 isolates (8.50%) were fungi. The main pathogens were *Klebsiella pneumoniae* (22.22%, 34/153), followed by *Escherichia coli* (11.76%, 18/153), *Staphylococcus aureus* (9.15%, 14/153) and *Acinetobacter baumannii* (8.50%, 13/153). In addition, the results showed that a total of 26 isolates (16.99%) were multidrug resistant bacteria, of which 13 isolates (50%) were carbapenem-resistant Enterobacteriaceae, 8 isolates (30.77%) were carbapenem-resistant *Acinetobacter baumannii*, 3 isolates (11.54%) were methicillin-resistant *Staphylococcus aureus*, and 2 isolates (7.69%) were carbapenem-resistant *Pseudomonas aeruginosa*. We found that 73.08% (19/26) of the multidrug-resistant bacteria were isolated from neurosurgery patients.

**Table 2:** Distribution and ratio of pathogens.

**Pathogens**	**Isolates (*N* = 153)**	**Ratio (%)**
**Gram-negative bacteria**	98	64.05
Klebsiella pneumoniae	34	22.22
Escherichia coli	18	11.76
Acinetobacter baumannii	13	8.50
Pseudomonas aeruginosa	8	5.23
Stenotrophomonas maltophilia	5	3.27
Other enterobacteria	20	13.07
**Gram-positive bacteria**	42	27.45
Staphylococcus aureus	14	9.15
Staphylococcus hemolyticus	4	2.61
Staphylococcus epidermidis	5	3.27
Streptococcus	7	4.58
Enterococcus	12	7.84
**Fungi**	13	8.50
Candida albicans	8	5.23
Other candida	5	3.27

### Univariate Analysis

Significant risk factors associated with postoperative pneumonia on univariate analysis are presented in Table 3. We found 25 perioperative risk factors that were significantly associated with postoperative pneumonia (*P* < 0.05), as follows: age, sex, BMI, smoking, drinking, hypertension, diabetes, malignancy, COPD, coma (GCS < 8), Surgical difficulty classification criteria, duration of surgery, emergency surgery, night operation, intraoperative blood loss, ASA physical status, duration of ventilation, deep vein catheterization, gastric tube intubation, amount of intraoperative liquid input, intraoperative RBC transfusion, postoperative hemoglobin level, postoperative albumin level and duration of postoperative bed rest.

**Table 3:** Risk factors for POP—univariate analysis.

**Risk factors**	**Cases (*N* = 264)**	**Controls (*N* = 264)**	***P*** **value**
Age (yr)	54.70 ± 14.85	48.73 ± 14.86	<0.001
Age grading			
≥60 yr	102 (38.64%)	59 (22.35%)	<0.001
<60 yr	162 (61.36%)	205 (77.65%)	
Sex			
Male	155 (58.71%)	132 (50.0%)	0.044
Female	109 (41.29%)	132 (50.0%)	
Weight (kg)	61.12 ± 10.88	60.35 ± 10.25	0.408
BMI			
≥24 kg/m2	103 (46.8%)	92 (35.4%)	0.011
<24 kg/m2	117 (53.2%)	168 (64.6%)	
ASA physical status			
≥ 3	143 (54.2%)	106 (40.2%)	0.001
< 3	121 (45.8%)	158 (59.8%)	
Coma (GCS < 8)	34 (12.9%)	5 (1.9%)	<0.001
Smoking	112 (42.4%)	83 (31.4%)	0.009
Drinking	91 (34.5%)	66 (25.0%)	0.017
Hypertension	81 (30.7%)	52 (19.7%)	0.004
Diabetes	28 (10.6%)	14 (5.3%)	0.024
Malignancy	86 (32.6%)	52 (19.7%)	0.001
COPD	33 (12.5%)	6 (2.3%)	<0.001
Coronary heart disease	7 (2.7%)	6 (2.3%)	0.779
Stroke	15 (5.7%)	8 (3.0%)	0.136
Liver disease	7 (2.7%)	10 (3.8%)	0.460
Renal dysfunction	6 (2.3%)	2 (0.8%)	0.154
Preoperative prophylactic antimicrobial use	254 (96.2%)	257 (97.3%)	0.460
Preoperative hemoglobin			
< 100 g/L	19 (7.2%)	15 (5.7%)	0.478
≥ 100 g/L	245 (92.8%)	249 (94.3%)	
Postoperative hemoglobin			
< 100 g/L	87 (33.0%)	44 (16.7%)	<0.001
≥ 100 g/L	177 (67.0%)	220 (83.3%)	
Preoperative albumin			
<35 g/L	47 (17.8%)	40 (15.2%)	0.412
≥35 g/L	217 (82.2%)	224 (84.8%)	
Postoperative albumin			
<35 g/L	198 (75.0%)	117 (44.3%)	<0.001
≥35 g/L	66 (25.0%)	147 (55.7%)	
Surgical difficulty classification criteria			
≥ 4	182 (68.9%)	151 (57.2%)	0.005
<3	82 (31.1%)	113 (42.8%)	
Duration of surgery (h)	4.67 ± 3.20	3.40 ± 2.18	<0.001
≥ 3 h	178 (67.4%)	132 (50.0%)	<0.001
< 3 h	86 (32.6%)	132 (50.0%)	
Emergency surgery	58 (22.1%)	19 (7.2%)	<0.001
Night operation	66 (25.0%)	12 (4.5%)	<0.001
Intraoperative blood loss (ml)	586.1 ± 1,428.0	243.2 ± 256.6	<0.001
≥ 400 ml	107 (40.5%)	67 (25.4%)	<0.001
<400 ml	157 (59.5%)	197 (74.6)	
Intraoperative RBC transfusion	48 (18.2%)	19 (7.2%)	<0.001
Intraoperative albumin infusion	16 (6.1%)	21 (8.0%)	0.394
Amount of intraoperative liquid			
≥ 4,000 ml	104 (39.4%)	56 (21.2%)	<0.001
<4,000 ml	160 (60.6%)	208 (78.8%)	
Radial artery cannulation	239 (90.5%)	233 (88.3%)	0.396
Deep vein catheterization	196 (74.2%)	148 (56.1%)	<0.001
Gastric tube intubation	84 (31.8%)	52 (19.7%)	0.001
Duration of ventilation			
≥ 24 h	81 (30.7%)	16 (6.1%)	<0.001
<24 h	183 (69.3%)	248 (93.9%)	
Duration of postoperative bed rest			
≥ 3 days	177 (67.0%)	86 (32.6%)	<0.001
<3 days	87 (33.0%)	178 (67.4%)	

*BMI, Body mass index; ASA, American Society of Anaesthesiologists physical status classification; GCS, Glasgow Coma Scale; COPD, chronic obstructive pulmonary disease; RBC, red blood cell*.

### Multivariate Regression Analysis

To further identify the independent risk factors for postoperative pneumonia, multivariate logistic regression analysis was performed using the 25 factors significantly associated with postoperative pneumonia in the univariate analysis. The results of the multivariate analysis are presented in Table 4. We found that there were six independent risk factors for postoperative pulmonary disease, as follows: increased age (*P* = 0.047, OR = 1.622, 95% CI: 1.006–2.614), COPD (*P* = 0.001, OR = 5.521, 95% CI: 2.093–14.565), emergency surgery (*P* = 0.004, OR = 3.407, 95% CI: 1.487–7.804), postoperative reduced albumin (*P* < 0.001, OR = 2.226, 95% CI: 1.447–3.423), prolonged mechanical ventilation (*P* = 0.047, OR = 1.949, 95% CI: 1.008–3.766), and longer duration of bed rest (*P* < 0.001, OR = 2.671, 95% CI: 1.694–4.212).

**Table 4:** Results of the multivariate analysis of factors associated with POP.

**Risk factors**	**B**	**S.E**.	**Wald**	***P*** **value**	**OR**	**OR 95%CI**
Age (≥ 60 yr)	0.483	0.244	3.940	0.047	1.622	1.006, 2.614
COPD	1.709	0.495	11.919	0.001	5.521	2.093, 14.565
Emergency surgery	1.226	0.423	8.401	0.004	3.407	1.487, 7.804
Postoperative albumin (<35 g/L)	0.800	0.220	13.281	<0.001	2.226	1.447, 3.423
Duration of ventilation (≥ 24 h)	0.667	0.336	3.938	0.047	1.949	1.008, 3.766
Duration of bed rest (≥ 3 days)	0.983	0.232	17.892	<0.001	2.671	1.694, 4.212

### Outcomes

The outcomes of the patients with postoperative pneumonia were retrospectively analyzed and are presented in Table 5. The results show that the hospital stay of patients in the case group (24.32 ± 14.64) was significantly longer than that in the control group (16.16 ± 8.36). In the case group, the proportion of patients with hospital stays over 14 days or 30 days were significantly higher than those in the control group (*P* < 0.05). In addition, the postoperative ICU occupancy rate, re-operation rate and postoperative mortality rate of patients in the case group were significantly higher than those in the control group (*P* < 0.05).

**Table 5:** The outcomes of postoperative pneumonia.

**Outcomes**	**Cases (*N* = 264)**	**Controls (*N* = 264)**	***P*** **value**
The hospital stay (days)	24.32 ± 14.64	16.16 ± 8.36	<0.001
≥ 14 days	202 (76.5%)	154 (58.3%)	<0.001
≥ 30 days	69 (26.1%)	14 (5.3%)	<0.001
ICU occupancy rate	123 (46.6%)	49 (18.6%)	<0.001
Re-operation rate	92 (34.8%)	7 (2.7%)	<0.001
Mortality rate	8 (3.0%)	0 (0%)	0.004

## Discussion

Among the 17,190 surgical patients included in this study, 264 cases of postoperative pneumonia occurred, with an incidence of 1.54%, which is similar to previously reported rates (5–7, 10). The incidence of postoperative pneumonia in neurosurgery (17.00%) was significantly higher than that in other surgical specialties. Obviously, neurosurgery patients are susceptible to pneumonia after surgery. Such patients usually suffer from complicated diseases and long coma, impairing their respiratory and immune function. In addition, the neurosurgery (102 cases), hepatobiliary (52 cases), general and digestive (35 cases), and thoracic (26 cases) were the four surgical specialties with the highest frequency of postoperative pneumonia, accounting for approximately 81.44% of the total. This suggests that it is particularly important to strengthen the management of patients at risk of postoperative pneumonia in these four surgical specialties.

The main pathogens were *Klebsiella pneumoniae* (22.22%), followed by *Escherichia coli* (11.76%), *Staphylococcus aureus* (9.15%) and *Acinetobacter baumann* (8.50%), accounting for half of the total pathogens. These four pathogens are conditional pathogenic bacteria that usually exist in the hospital environment and oropharynx of patients. Obviously, surgical trauma destroys the integrity of the body's skin and tissues, damaging the patient's first line of immune defense and providing opportunities for these bacterial infections. Meanwhile, *Staphylococcus aureus* can be transmitted by hand contact, indicating the need for strict hand hygiene prior to invasive procedures such as tracheal intubation. The drug resistance of pathogens has also become a serious problem, which makes clinical treatment more difficult. In this study, the incidence of multidrug-resistant bacteria was approximately 16.99%, of which 73.08% occurred in neurosurgery patients.

This study has demonstrated that age over 60 years old is an independent risk factor for postoperative pneumonia, which is consistent with previous research (11–13). Kunisaki C et al. (11) reported a significant variation in the postoperative pneumonia rate between patients aged over 75 years and those aged 45–65 years (13.3% and 6.3%). Furthermore, Yamada H et al. (12) reported that the postoperative pneumonia incidence in patients aged over 85 years was significantly higher than that in patients aged 75 to 85 years (16.7% and 3.3%). These two studies showed that the risk of postoperative pneumonia increased significantly with patient age, which was also confirmed by Miki Y et al. (13).

Our study found that the incidence of postoperative pneumonia in patients with COPD was 4.5 times greater than that in unaffected patients (*P* = 0.001, OR = 5.521, 95% CI: 2.093–14.565). Pulmonary chronic inflammation in patients with COPD is a characteristic pathological change that will continue to destroy the alveolar wall septum and result in pulmonary interstitial fibrosis (9, 14). Some previous studies have confirmed that preoperative treatment for COPD can reduce the incidence of postoperative pneumonia (15, 16). Numata T et al. (15) have demonstrated that long-acting anticholinergic drugs and long-acting β2 receptor agonists can effectively reduce the rate of postoperative pulmonary complications in patients with COPD. Du Z et al. (16) have further demonstrated that perioperative aerosol inhalation of ipratropium bromide can reduce the incidence of postoperative pneumonia in COPD patients undergoing thoracic surgery.

We also found that the rate of postoperative pneumonia in emergency surgery patients was 2.4 times greater than that in non–emergency surgery patients (*P* = 0.004, OR = 3.407, 95% CI: 1.487–7.804), which is similar to the results reported by Kim Th et al. (17). Due to the urgency of the surgery, the preoperative preparations appear to be particularly poor, and the prevention of infection is usually not strict enough. Furthermore, McCoy CC et al. (18) reported that compared with elective surgery, emergency surgery was associated with an increased risk of serious postoperative complications and increased the risk of postoperative death by approximately 1.39 times.

The serum albumin level is the most common indicator used to evaluate the nutritional status of patients. A serum albumin level below 35 g/L is generally considered malnutrition and has been identified as a potential risk factor for poor postoperative outcomes (19, 20). Our findings suggest that postoperative albumin levels under 35 g/L in surgical patients is an independent risk factor for postoperative pneumonia. A decrease in postoperative albumin can directly reflect that the metabolism of the body is in a negative nitrogen balance and engaged in high protein consumption. The results of univariate analysis in our study showed that intraoperative albumin infusion did not increase the risk of postoperative pneumonia, indicating that a timely albumin infusion can be applied to correct hypoalbuminemia if necessary.

Our study demonstrated that the duration of mechanical ventilation over 24 h was an independent risk factor for postoperative pneumonia (*P* = 0.047, OR = 1.949, 95% CI: 1.008–3.766). For patients sent to the ICU after surgery, the duration of ventilation usually exceeds 24 h. Thus, extubation timely after surgery can significantly reduce the incidence of postoperative pneumonia. Vera Urquiza R et al. (21) found that extubation 6 h later was an independent risk factor for postoperative pneumonia (*P* = 0.005, OR: 15.81, 95% CI: 2.2–110.7). Savardekar A et al. (22) confirmed that endotracheal intubation for more than 48 h was an independent risk factor for pneumonia (*P* = 0.041, OR = 6.638, 95% CI: 1.08–40.8).

Duration of bed rest after surgery over 3 days was an independent risk factor for postoperative pneumonia (*P* < 0.001, OR = 2.671, 95% CI: 1.694–4.212).

Therefore, patients ought to start off-bed activity early after surgery if there is no special contraindication. When it is necessary to stay in bed for a long time, the patient should be encouraged to expectorate regularly and clear their airway secretions. Cassidy MR et al. (23) implemented a multidisciplinary team cooperation model, proving that early out of bed after surgery can effectively reduce the occurrence of postoperative pneumonia.

Furthermore, we found that postoperative pneumonia usually caused poor outcomes. The hospital stay of cases (24.32 ± 14.64) was significantly longer than that of the matched controls (16.16 ± 8.36), and postoperative pneumonia significantly increased the ICU occupancy rate, reoperation rate, and perioperative mortality rate. A retrospective analysis of 1,415 consecutive gastric cancer patients reported that postoperative pneumonia was associated with poor long-term outcomes (24). Fujishima S et al. (25) found that postoperative pneumonia in patients with esophageal cancer was associated with skeletal muscle consumption and asymptomatic pneumonia within 6 months after surgery, and the survival time of patients with postoperative pneumonia was significantly lower than that of patients without pneumonia. Obviously, the real value of study is in improving quality of life (26).

A formal statement of shortcomings could keep authors and the public from overstating a study's claims (27). As a single-center study, the external validity of the findings is limited. Due to the retrospective collection of most clinical data, information on the exposures is subject to observation bias. Specifically, although our diagnostic criteria are very specific, our researchers may still make diagnostic errors, even in the same patient. In addition, the rate of postoperative pneumonia varied greatly in each surgical department, and the representation of patients included in the study is prone to bias. Many surgical patients receive prophylactic antibiotics, which tends to skew the study results and obscure the risk factors associated with postoperative pneumonia. Furthermore, this study did not follow up on the long-term outcomes of patients with postoperative pneumonia.

## Conclusions

Postoperative pneumonia is associated with severe clinical outcomes. In this retrospective single-center study, we identified six independent risk factors that can aid in risk stratification and management of patients at risk of postoperative pneumonia. The distribution of causative pathogens can also help in the implementation of effective preventions and interventions, which has great implications for the formulation of infection control policies.

## Data Availability Statement

The raw data supporting the conclusions of this article will be made available by the authors, without undue reservation.

## Author Contributions

BX collected, analyzed and interpreted all data, and was a major contributor in writing the manuscript. YS participated in study design and data analysis. FY was involved in data acquisition and analysis. RC was involved in data acquisition and manuscript writing. YY conducted statistics and analysis of data. SJ guided paper writing and provided financial and technical support. All authors read and approved the final submitted manuscript. All authors contributed to the article and approved the submitted version.

## Funding

Project supported by the Applied Basic Research Foundation of Yunnan Province (CN) (2014FZ027). The funder was not involved in the design of the study and collection, analysis, and interpretation of data and in writing the manuscript.

## Conflict of Interest

The authors declare that the research was conducted in the absence of any commercial or financial relationships that could be construed as a potential conflict of interest.

## Publisher's Note

All claims expressed in this article are solely those of the authors and do not necessarily represent those of their affiliated organizations, or those of the publisher, the editors and the reviewers. Any product that may be evaluated in this article, or claim that may be made by its manufacturer, is not guaranteed or endorsed by the publisher.

## Abbreviations

POP: postoperative pneumonia
HAP: hospital acquired pneumonia
BMI: body mass index
GCS: Glasgow Coma Scale
ASA: American Society of Anesthesiologists
COPD: chronic obstructive pulmonary disease
Hb: hemoglobin
RBC: red blood cells
MDR: Multi-drug-resistance
ICU: Intensive Care Unit
OR: odds ratio
CI: confidence interval.
